# Seropositivity of Toxoplasmosis in Antenatal Women with Bad Obstetric History in a Tertiary-care Hospital of Andhra Pradesh, India

**DOI:** 10.3329/jhpn.v30i1.11287

**Published:** 2012-03

**Authors:** Munmun Das Sarkar, B. Anuradha, Neelam Sharma, Rabindra Nath Roy

**Affiliations:** ^1^Burdwan Medical College, Burdwan, West Bengal, India; ^2^Mamata Medical College, Khammam, Andhra Pradesh, India

**Keywords:** Bad obstetric history, Case-control studies, Descriptive studies, Seroprevalence, *Toxoplasma gondii*, Toxoplasmosis, India

## Abstract

Toxoplasmosis is a well-documented cause of bad obstetric history (BOH) and a major reason of congenitally-acquired infection. The study was conducted to determine the seropositivity of toxoplasmosis in women with BOH, attending the antenatal clinic of the Mamata General Hospital, Khammam, Andhra Pradesh, India. The study subjects included 105 antenatal women with BOH and 105 antenatal women who had previous normal deliveries. A serological evaluation was carried out to determine the presence of *Toxoplasma gondii*-specific IgG and IgM antibodies, using commercial diagnostic kits, by the enzyme-linked immunosorbent assay method. The seropositivity for *Toxoplasma* was 49.52% in the study group compared to 12.38% in the control group. The difference in seropositivity was significant (p=0.00). The seroprevalence gradually increased with advancing age. Abortion (51.92%) was the commonest form of pregnancy wastage, followed by stillbirths (36.53%) and premature deliveries (7.69%). The seropositivity of toxoplasmosis was significantly higher in the study group than that in the control group, and the seropositivity played an important role in determining the foetal outcome. Considering the subclinical pattern of infection, routine serological test is recommended for all pregnant women for both IgG and IgM antibodies.

## INTRODUCTION

Toxoplasmosis is caused by an obligate intracellular protozoan parasite *Toxoplasma gondii* (1). It is one of the most common parasitic infections of humans and other warm-blooded animals. Approximately one-third of the population are exposed to this parasite (2). Infection in an immunocompetent adult human is usually asymptomatic. Toxoplasmosis is most dangerous for immunocompromised patients and the foetus whose mother is infected during pregnancy (3).

Transmission of the infection to the foetus usually occurs when primary infection is acquired by an immunologically-normal mother during gestation (4). Infected pregnant women are often asymptomatic or many have only mild symptoms, making the diagnosis difficult (1,3). The organism is transmitted haematogenously to the placenta. When this occurs, infection may be transmitted to the foetus transplacentally or during vaginal delivery (4). The risk of congenital toxoplasmosis depends on the timing of the mother's acute infection. The disease is usually severe when acquired in the first trimester, and the severity decreases as the gestational age advances, leading to a mild or an inapparent disease at birth. The different rates of transmission and outcomes are most likely related to placental blood-flow, the virulence and amount of *T. gondii* acquired, and the immunologic ability of the mother to restrict parasitaemia (4). The overall rate of transmission of maternal infection to the foetus is about 45%. Of these, 60% are subclinical infections, 9% resulting in death of the foetus, and 30% have severe damage (5).

The clinical implications of infection due to *Toxoplasma* in pregnant patients are manifold. Such patients may have spontaneous abortions, stillbirths, intrauterine growth retardation, preterm deliveries, or foetal anomalies (6).

Various manifestations of congenital infection may occur in the perinatal period. These range from relatively-mild signs, such as prematurity, peripheral retinal scars, sensory deficits, developmental delay, impaired psychomotor performance, and mental retardation to the classic triad of signs consisting of hydrocephalus, intracerebral calcification, and chorioretinitis (4,7).

Toxoplasmosis is, thus, a significant risk factor for bad obstetric outcome and a major cause of congenitally-acquired infection, leading to a high degree of intrauterine foetal death and morbidity of the newborn. Treatment of a pregnant woman who acquires infection at any time during pregnancy reduces the chance of congenital infection in her infant by approximately 60% (4). Therefore, early diagnosis of toxoplasmosis is essential to start appropriate treatment on time to reduce the transplacental transmission.

Toxoplasmosis is usually diagnosed by serological tests for *Toxoplasma*-specific IgM and IgG antibodies. A positive IgG titre is sufficient to establish that a patient has been infected with *T. gondii* but a negative IgM result virtually rules out a recently-acquired infection, unless sera are tested so early when an antibody response has not yet developed or is undetectable (2).

Against this background, we attempted to determine the extent of seropositivity of toxoplasmosis in antenatal women with bad obstetric history (BOH) and to compare it with that in antenatal women having previous normal obstetric history, attending the antenatal clinic of a tertiary-care hospital in Khammam, Andhra Pradesh, India.

## MATERIALS AND METHODS

### Study area

This descriptive case-control study was conducted in 2007 at the antenatal clinic of the Mamata General Hospital which is a super-specialty rural hospital attached to the Mamata Medical College, Khammam, Andhra Pradesh, India.

### Study subjects

The study group comprised antenatal women of reproductive age with previous BOH, including unfavourable foetal outcome in terms of abortions, intrauterine foetal death, stillbirths, preterm deliveries, unexplained early neonatal death, and congenital anomalies. The control group comprised multiparous, age-matched antenatal women without BOH attending the same clinic.

### Sample-size

To work out the required sample-size for the determination of seropositivity in antenatal women with BOH, the equation suggested by Vaughan and Morrow was used (8). Considering 49.47% seropositivi-ty of toxoplasmosis in women with BOH (6), 10% margin of sampling error, and 1% non-response rate, the desired sample-size was 105. About 2,210 pregnant women attended the antenatal clinic of the hospital in the previous year. Of these women, 125 presented with BOH. Considering the admission trend, the investigators expected to obtain the desired sample within 10 months. In total, 210 women were included in the study―105 with BOH (study group) and 105 without BOH (control group).

### Collection of data

The designated investigators visited the outpatient department on alternate day, selected the study subjects, and screened them using a predesigned pretested schedule considering the inclusion and exclusion criteria till 105 study subjects could be identified. The next available age-matched multiparous antenatal woman without BOH was included in the control group subjects.

Clinical examination and laboratory investigations were carried out for the study subjects to exclude other causes of foetal wastage, such as hypertension, diabetes mellitus, syphilis, Rh (rhesus) incompatibility, physical causes of abortion, and consanguinity. Subjects with known causes of foetal wastage were excluded from the study. All of them were interviewed to ascertain age, medical and obstetric information.

For serological analysis, 2 mL of venous blood was collected in a sterile container with strict aseptic precautions from each study subject. The serum was separated and stored in numbered aliquots at −20 °C till assayed. All the serum samples collected from the study and control groups were tested for *Toxoplasma* IgM and IgG antibodies by commercially-available (ELISA) kits (98% sensitivity, 98% specificity) (Equipar Diagnostici, via G. Ferrari, 21/N 21047 Saronno (Va), Italy). The results were read by a Microwell reader and compared in a parallel manner with controls; optical density was read at 450 nm on an ELISA reader.

### Analysis of data

Collected data were compiled in Microsoft Excel spreadsheet. The proportion and the mean value were computed in appropriate situations. To find out any association between categorical data, chi-square test was employed using the Epi Info software (version 3.5.1).

### Ethical approval

The ethical committee of the concerned institute approved the research protocol. The purpose and procedures of the study were explained to all the study subjects, and informed consent was obtained from them.

## RESULTS

### Seropositivity among study and control groups

Table 1 shows that 49.52% were seropositive cases in the study group and 12.38% in the control group. The seropositivity of IgG alone was observed in 27.61% and 9.52% of the study and the control group subjects respectively. In the study group, 21.9% of the subjects were positive for both IgG and IgM antibodies while it was 2.86% in the control group. None was positive for IgM antibody alone. The difference in seropositivity was significant (p=0.00).

**Table 1.** Seropositivity among study and control groups*

Group	No. of cases	No. (%) of cases positive for IgG only	No. (%) of cases positive for both IgG and IgM	Total no. (%) of seropositive cases
Study	105	29 (27.61)	23 (21.9)	52 (49.52)
Control	105	10 (9.52)	3 (2.86)	13 (12.38)

*None of the subjects was positive for IgM only in both the groups;

Odds ratio=6.94;

χ²=33.89 (among total seropositive cases);

df=1, p=0.00;

df=Degree of freedom

### Seropositivity in relation to age of subjects

The seropositivity was analyzed in relation to the age of the study subjects (Table 2). It was observed that the seropositivity increased with advancing age in both the groups. The seropositivity rate varied from 25% to 60.71% in the study group and from 0% to 17.64% in the control group. The z-test was applied to examine the significance of difference in the proportion of seropositivity between the two groups. The differences were not significant for the age-group of up to 25 years; however, the differences were significant for the age-groups of above 25 years.

### Seropositivity in relation to number of bad obstetric outcome

The seropositivity of *Toxoplasma* in relation to the number of times the antenatal woman experienced BOH revealed that the highest percentages (52.38%) of positivity was noted among women with a history of two BOH, followed by 50% positivity among women with three or more BOH, and the lowest (47.27%) among women having one BOH (Table 3). The difference was not significant (p=0.88).

**Table 2.** Seropositivity in relation to age of subjects

Age (years)	Study group			Control group			z and p value
	Sera tested	Positive sera		Sera tested	Positive sera		
		No.	%		No.	%	
≤20	12	3	25.00	7	0	0.00	0.60 (0.27)
21-25	26	10	38.46	46	5	10.86	0.89 (0.18)
26-30	39	22	56.41	35	5	14.28	2.56 (0.00)
>30	28	17	60.71	17	3	17.64	2.04 (0.02)

### Seropositivity in relation to type of bad obstetric history

The seropositive cases were distributed in relation to the type of BOH (Table 4). It revealed that abortion (51.92%) was the commonest form of pregnancy wastage, followed by stillbirths (36.53%), premature deliveries (7.69%), congenital anomalies (1.93%), and early unexplained neonatal deaths (1.93%).

## DISCUSSION

Toxoplasmosis is a zoonotic disease with worldwide distribution. Most *Toxoplasma*-associated infections among people occur by ingesting tissue cysts from infected meat or oocysts from soil or by congenital transmission through the placenta. The seropositivity levels vary widely in different regions of the globe. The rates of prevalence change according to the nutritional factors, sociocultural habits, geographic factors, climate, and transmission route and typically rise with age (9).

Interpretation of *Toxoplasma* serology was done according to Burrow and Duffy (10); when a positive IgM test result indicates acute/early infection, IgG titres should be retested in several weeks. A positive IgG titre is sufficient to establish that a patient has been infected with *T. gondii*. Recent infection is indicated by a positive test result for both IgG and IgM, and remote infection is usually indicated by a negative IgM with the positive IgG test result.

**Table 3.** Seropositivity in relation to number of bad obstetric outcomes

No. of times with bad obstetric outcome	No. of sera tested	Seropositive	No. %	Seronegative	No. %
1	55	26	47.27	29	52.72
2	42	22	52.38	20	47.61
3 and more	8	4	50.00	4	50.00

χ²=0.25;

p=0.88;

df=1;

df=Degree of freedom

The prevalence of this infection reported in different studies in India shows a wide variation, and one pilot study on women in Kumaon region in 1991 reported the prevalence of 77%, the highest reported so far in India (11). A recent pilot study in the same region in 2005 reported 55% for *Toxoplasma* IgG antibodies and 20% for IgM antibodies (12). Singh *et al*. found an overall anti-*Toxoplasma* IgG seroprevalence of 45% among pregnant women in New Delhi (13).

We analyzed the odds ratio (6.94) to estimate the risk of adverse pregnancy outcome associated with exposures to toxoplasmosis. This suggests a possible causal relationship between *T. gondii*-associated infection and BOH. In our study, the overall seroprevalence of toxoplasmosis was 49.52% among antenatal women with BOH compared to 12.38% in the control group (Table 1). The compatible seropositivity of 49.47% in the study group and 8.88% in the control group was reported by Zargar *et al*. (6). Borkakoty *et al*. reported the seroprevalence of 44.6% and 36.8% among the study and the control group respectively (14). However, Singh and Dangvela observed an overall incidence of 13.3% in the study group and 4.0% in the control group (15). Results of a study by Bachhiwal *et al*. showed that the overall seroprevalence of toxoplasmosis was 22% (55/250) among study subjects. In the control group, it was 2% (1/50). The difference was also significant (16).

In the present study, 27.61% of the women were positive for IgG only, indicating remote infection, and 21.9% were positive for both IgG and IgM, indicating recent infection. A similar result was reported from a study in Hyderabad in 2001. Of 13 serum samples screened, the positivity for IgG was 43.3%, and the positivity for both IgG and IgM was 32% (17). The same investigators performed another similar study in 2004 and analyzed 100 serum samples, which revealed that 33%, 5%, and 18.2% of samples were positive for IgG, IgM, and both IgG and IgM respectively (18). A study in Manipur also recorded the seropositivity of 58.14% for IgG, 2.32% for IgM, and 6.97% for both IgG and IgM (19).

In the control group of the present study, the seropositivity of IgG, indicating remote infection, was 9.52%, and it was 2.86% for both IgG and IgM, indicating recent infection (Table 1). Sandhu *et al*. detected 8% IgG seropositivity among antenatal women without BOH, and none of them was positive for IgM (20). Borkakoty *et al*. also observed the IgG seroprevalence of 36.8% among pregnant women without history of pregnancy wastage; in addition, the IgM seroprevalence was 5.9% among the same group (14).

The percentage of seropositivity of toxoplasmosis demonstrated a gradual increase with advancing age of the mother, and the percentage was the highest among women of age-group of >30 years in our study (Table 2). Spalding *et al*. also observed a higher prevalence among people aged 20-30 years, and the prevalence of infection was increasing with older age-group (21). Mittal *et al*. opined that the seropositivity of *Toxoplasma* increases with age. According to their study, the lowest rate of antibody acquisition occurred in the age-group of 15-20 years; it increased with the increase in age; and the maximum prevalence was observed among women aged 36 years and above (22). Borkakoty *et al*. have also shown that the higher prevalence of infection due to *T. gondii* was associated with increase in age (14). This may be due to the fact that the frequency of infection increases with older age-groups as the probability of an individual coming into contact with the transmission routes increases as his or her age increases (21).

**Table 4.** Seropositivity in relation to type of bad obstetric history

Type of bad obstetric history	Seropositive (n=52)	%(n=52)
Abortion	27	51.92
IUFD/stillbirth	19	36.53
Premature delivery	4	7.69
Abortion and congenital defects*	1	1.93
Unexplained neonatal death	1	1.93

*First pregnancy–abortion and second pregnancy–congenitally-malformed liveborn baby;

IUFD=Intrauterine foetal death

In the present study, the percentage of seropositivity was also assessed in relation to the number of BOH. Table 3 clearly shows that there was no relationship between increase in the number of pregnancy wastages and seropositivity. This corroborates the observation of Borkakoty *et al*. (14). According to their study, the increase in the number of pregnancy wastages had no significant association with infection due to *T. gondii* (14). Women of childbearing age, with no previous exposure to *Toxoplasma,* are at a risk of acquiring acute infection during pregnancy, resulting in congenital toxoplasmosis (3). Women give birth to children with congenital infection only once (23). If the mother was infected by the parasite before the current pregnancy, babies do not suffer from congenital toxoplasmosis. Transplacental transmission from chronic infection does not occur (1).

Among the seropositive cases, abortion (51.92%) was the commonest form of pregnancy wastage, followed by stillbirths/intrauterine foetal deaths (36.53%), premature deliveries (7.69%), congenital anomalies (1.93%), and early unexplained neonatal deaths (1.93%). Surpam *et al*. observed abortions in 27.27%, intrauterine growth restriction in 9.37%, intrauterine foetal death in 17.64%, and preterm labour in 18.18% of cases (24). Kandle *et al*. reported similar results in their study; 42 (50%) abortion cases were positive for anti-*Toxoplasma* antibodies (25). The result also partially matched with the observations of Bachhiwal *et al*. who reported that abortion was the commonest (85.45%) form of pregnancy wastage, followed by congenital anomalies, stillbirths, and premature deliveries (16). However, the observation differed from that of Saxena *et al*. According to them, positivity was maximum in cases with history of congenital malformations (33.3%), followed by cases with history of premature labour, abortions, and stillbirths (26). As such, the seroprevalence of toxoplasmosis was observed more among patients with BOH. Rai from Nepal examined 273 patients with various medical ailments and found that, of different groups, women with BOH had the highest seropositivity of *Toxoplasma* (27).

### Limitations

Several potential limitations should be considered in interpreting the results of the present study. First, this is a hospital-based study in a rural set-up, which may not be representative of and applicable to the general population. Second, due to some constrains, we could not perform the IgG avidity test, which is needed to differentiate between acute/recent and past infections in cases showing seropositivity for both IgM and IgG. Moreover, this is the first study on toxoplasmosis from this area. No previous data were available for reference. It can, however, provide a clear snapshot of the current situation, provide a reasonably precise and reliable estimate of the magnitude of risk, and may help improve the management and design of future studies to explore further.

### Conclusions

The present study is directed at providing the profile of the seroprevalence of toxoplasmosis among antenatal women with and without BOH among the study subjects from Khammam, Andhra Pradesh, India. The findings revealed that the seropositivity of toxoplasmosis was significantly higher in the study group than that in the control group. There was a gradual increase in seroprevalence with advancing age, and the maximum seroprevalence was observed in the age-group of >30 years. The maximum seropositivity was observed in abortion cases.

The results indicate that toxoplasmosis is quite prevalent in and around Khammam. It is a well-documented risk factor for BOH and may play a vital role in determining the foetal outcome. As the majority of acquired infections are subclinical, there is a need for regular screening of all pregnant women for this infection. The initiation of judicious treatment in time can, thus, be provided to prevent morbidity and mortality of infants born to such mothers.

### *Recommendations*

Based on the findings of the study, we recommend the following steps for the appropriate management of this health problem:

- Routine serological testing is to be advised to all pregnant women with or without BOH, attending the antenatal clinics for both *Toxoplasma*-specific IgG and IgM antibodies.
- For women with negative results in both the tests, information and education relating to preventive measures are of paramount importance because they are at a high risk of acquiring a primary infection during pregnancy.
- Women who are showing both IgM and IgG-positive results, differentiation between recent infection and pre-existing infection may be done with the IgG avidity test and those showing low avidity be treated accordingly.
